# The study of n-3PUFAs protecting the intestinal barrier in rat HS/R model

**DOI:** 10.1186/1476-511X-13-146

**Published:** 2014-09-08

**Authors:** Yang Li, Xinying Wang, Ning Li, Jieshou Li

**Affiliations:** Research Institute of General Surgery, Jinling Hospital, School of Medicine, Nanjing University, 305 Zhongshan East Road, Nanjing, 210002 China

**Keywords:** Hemorrhagic shock, Resuscitation, Tight junction, Occludin protein, I-FABP, n-3 PUFAs

## Abstract

**Background:**

N-3 PUFAs have been demonstrated in vitro it could prevent the intestinal tight junctions (TJs) from the ischemia/re-perfusion injury and the inflammatory reaction injury. The purpose of this study was to evaluate the protection of n-3 PUFAs on the intestinal TJs in the rat model of hemorrhagic shock followed by resuscitation.

**Methods:**

Male SD rats (n = 72; 250 ~ 300 g) were randomly divided into 6 groups: SHAM, hemorrhagic shock (HS), hemorrhagic shock/resuscitation (HS/R), ω-6 group, ω-3 group and ω-3 treatment group. Shock was induced, and a mean arterial pressure was maintained at 35 to 40 mmHg for 60 minutes. Resuscitation was carried out by returning half of the shed blood and Ringer’s lactate solution. In ω-6 and ω-3 group, Intralipid or fish oil (0.2 g/Kg), respectively, was infused 30 minutes after shock. And fish oil was infused with resuscitation in ω-3 treatment group. Half of each group was killed at 30 minutes and 4 hours after resuscitation, respectively. The serum samples and the intestinal sample was collected for further examination.

**Result:**

There is no difference between ω-3, ω-3 treatment and sham group in Chiu’s score, but the other three groups have higher scores than they did. Compared with HS, HSR and ω-6 group, ω-3 and ω-3 treatment group showed most intact in intestinal mucoscal villi and TJs through HE, SEM and LSCM. The levels of IL-6 and TNF-α of bowel tissue in ω-3 and ω-3 treatment group were significantly lower than HS and HSR groups’. At the time point of 30 min, the levels of serum endotoxin were dramatically higher in HS、HSR and ω-6 groups when compared with ω-3, ω-3 treatment and sham group. However, it in ω-3 group was greater than sham and HS group until 4 hours.

**Conclusion:**

Fish oil pretreatment before resuscitation showed a beneficial effect to the intestinal TJs and atteunated inflammation after H/R in HS/R rat model and is better than ω-6 PUFAs did.

## Introduction

Hemorrhagic shock, an independent risk factor for multiple-organ dysfunction syndrome (MODS) [1], is the leading cause of death in traumatic patients. It followed by resuscitation is considered as an insult frequently induces a systemic inflammatory response syndrome (SIRS) that results in MODS, which is a major clinical problem. The concept has developed that the intestinal injury/resuscitation (I/R) injury associated with hemorrhagic shock (HS) followed by fluid resuscitation is responsible for the failure of the intestinal barrier, bacterial translocation, systemic sepsis, and even MODS [2]. Meanwhile, tight junctions (Tjs), serve as a fence between two cells, play a major role in this mechanism. Hypoperfusion or ischemia can cause disruption of the Tjs, with subsequent barrier failure. The loss of intestinal cell integrity leads to paracellular leakage of microbial products [3–6].

The influence of the n-3 polyunsaturated fatty acids (n-3 PUFAs) on inflammatory bowel disease indicated 50% reduction in steroid use and improved histology [7], delayed relapse [8], reduced disease activity and drugs usage [4]. Also, the influence of the n-3 fatty acids on patients under surgical stress indicated reduced wound and major infections, reduced intra-abdominal abscess and multiple organ failure, and shortened hospital stay in several prospective randomized studies in burn, trauma and major surgery patients. The benefit of n-3PUFAs on barrier barrier also was found in sever pancreatitis, trauma and septic shock. However, the effect of the n-3PUFAs on intestinal permeability in hemorrhagic shock/resuscitation (HS/R) is rarely reported [9].

So we make a hypothesis that intravenous n-3 PUFAs would improve intestinal barrier function in a rat model of hemorrhagic shock with resuscitation.

## Result

### Characteristics and shock preparation of rats

All animals survived from shock and completed resuscitation in the four groups. Among the groups, there was no difference in the change of blood pressure into baseline following shock and resuscitation (Table 1). Except for the sham group, all animals were maintained at 35 to 40 mm Hg for 60 minutes by periodic withdrawal or return of blood. There were no detectable differences between the four groups in terms of body weight, cannulation time, or amount of blood withdrawn.

**Table 1:** **Hemorrhagic shock parameters** ^**a**^

	sham	HS	HS/R	ω-6 group	ω-3 group	ω-3 Tre	P
Weight,(g)	232.4 ± 14.7	240.2 ± 16.4	223.3 ± 9.43	230.67 ± 11.0	230.8 ± 8.87	240.0 ± 10.8	NS
Cannulation time,(min)	20.13 ± 1.69	20.78 ± 1.40	20.60 ± 2.07	21.63 ± 1.25	19.12 ± 1.43	22.11 ± 3.61	NS
Blood removed, (ml)	5.34 ± 0.27	5.82 ± 0.23	6.15 ± 0.26	5.86 ± 0.64	6.03 ± 0.52	5.97 ± 0.49	NS

NS, not significant. ^a^Data are given as mean ± standard error of the mean.

### I-FABP and endotoxin in plasma

**Figure 1:**
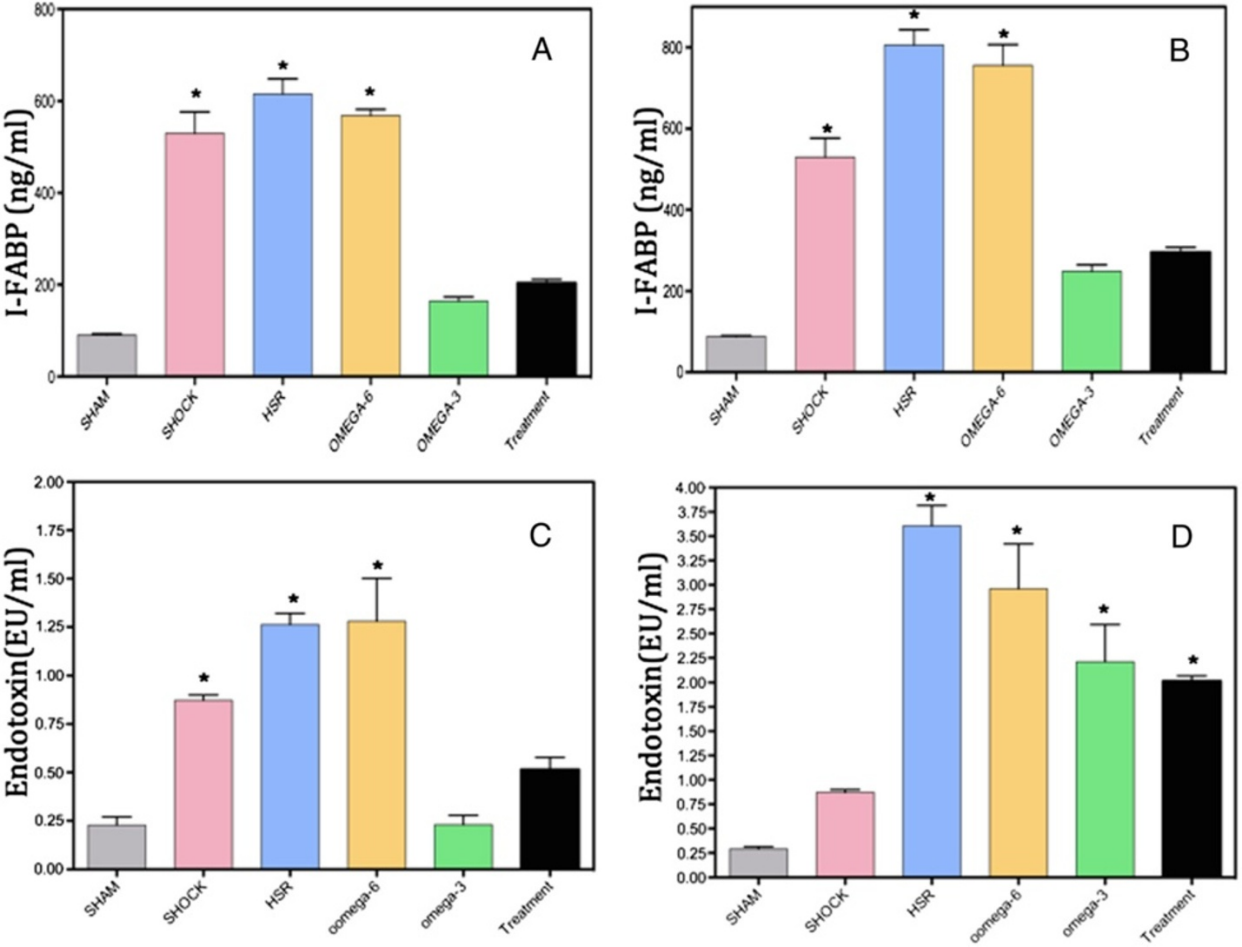
**The levels of I-FABP and endotoxin of plasma.** I-FABP in Sham group, ω-3 group and ω-3 treatment group were significantly lower than HS,HS/R and ω-6 group (p < 0.05). The datas in 30 min and 4 hours have the same trend **(A, B)**. The levels of endotoxin in ω-3 group and ω-3 treatment group were dramatically increased in 30 min when compared with 4 hours **(C, D)**.

HS/R group has the highest concentration of intestinal fatty acid binding protein (I-FABP) in all the groups. 30 min and 4 hours after resuscitation, the levels of I-FABP in ω-3 group and ω-3 treatment group had little change compared with sham group, but they were significantly lower than HS, HS/R and ω-6 group (p < 0.05). (Figure 1A, B)30 min after resuscitation, the levels of endotoxin in ω-3 group and ω-3 treatment group were remarkablely lower than HS, HS/R and ω-6 group (p < 0.05), however, they increased 4 hours although there were no differences compared with HS/R and ω-6 group, respectively. (Figure 1C, D)

### IL-6 and TNF-α in tissue

Interleukin-6 (IL-6) was measured 4 hours postresuscitation because it characteristically has a later response. Interestingly, the level of IL-6 were elevated in ω-3 group and ω-3 treatment group animals without significant difference compared with the sham group. But the levels of IL-6 in the other groups were elevated dramatically (Table 2).

**Table 2:** **Results of cytokine protein measurements (pg/mL tissue)**

	sham	HS	HS/R	ω-6 group	ω-3 group	ω-3 Tre
IL-6 (4 h)	35.23 ± 6.16	91.50 ± 33.62*	122.22 ± 41.28**	161.32 ± 19.31**	53.47 ± 25.81	71.44 ± 32.21
TNF-α (30 min)	30.84 ± 9.85	90.39 ± 33.76*	141.29 ± 75.43**	126.99 ± 19.89**	56.85 ± 17.46	88.12 ± 8.09

*P < 0.05. Data are given as mean ± standard error of the mean.

The concentration of tumor necrosis factor-α (TNF-α) was measured 30 minutes after shock and resuscitation because it is known to be characteristic of an early response to hemorrhagic shock. TNF-α was markedly elevated in HS, HS/R, ω-6 group and ω-3 treatment group (Table 2). However, there was no significant difference between the control and fish oil groups.

### Lipid analysis

Levels of docosahexenoic acid (DHA) and eicosapentenoic acid (EPA) were found to be significantly elevated in plasma in ω-3 group and ω-3 treatment group as compared with other groups, whereas levels of arachidonic acid (AA) were correspondingly reduced. The levels of DHA and EPA in intestinal epithelial cells had the same change trend, although there were no significant differences in among all groups (Tables 3 and 4).

**Table 3:** **Fatty acids in plasma (g/L)**

	sham	HS	HS/R	ω-6 group	ω-3 group	ω-3 Tre	P
EPA (c 20:5ω-3)	0.14 ± 0.00	0.11 ± 0.05	0.10 ± 0.02	0.15 ± 0.01	18.8 ± 5.7	19.1 ± 6.5	0.01
DHA (c 22:6ω-3)	3.5 ± 1.69	2.1 ± 0.74	2.0 ± 0.89	4.5 ± 0.25	16.4 ± 4.5	20.11 ± 3.61	0.01
AA (C20:4ω-6)	10.5 ± 3.17	11.2 ± 3.23	9.1 ± 4.01	21 ± 4.61	9.20 ± 3.42	10.97 ± 5.39	0.01

Data are given as mean ± standard error of the mean. AA, arachidonic acid; DHA, docosahexaenoic acid; EPA, eicosapentaenoic acid.

**Table 4:** ^**a**^ **Fatty acids in intestinal epithelial cells**

	sham	HS	HS/R	ω-6 group	ω-3 group	ω-3 Tre	P
EPA (c 20:5ω-3)	2.01 ± 0.45	1.98 ± 0.12	2.10 ± 0.25	2.41 ± 0.51	5.47 ± 1.21	5.23 ± 1.5	NS
DHA (c 22:6ω-3)	2.16 ± 0.78	1.59 ± 0.44	2.50 ± 0.49	3.12 ± 0.75	6.45 ± 0.23	5.10 ± 1.45	NS
AA (C20:4ω-6)	15.3 ± 4.15	16.2 ± 3.63	17.1 ± 4.54	25 ± 5.46	17.5 ± 4.44	16.7 ± 3.72	NS

NS, not significant. ^a^Data are given as mean ± standard error of the mean. AA, arachidonic acid; DHA, docosahexaenoic acid; EPA, eicosapentaenoic acid.

### Histology

**Figure 2:**
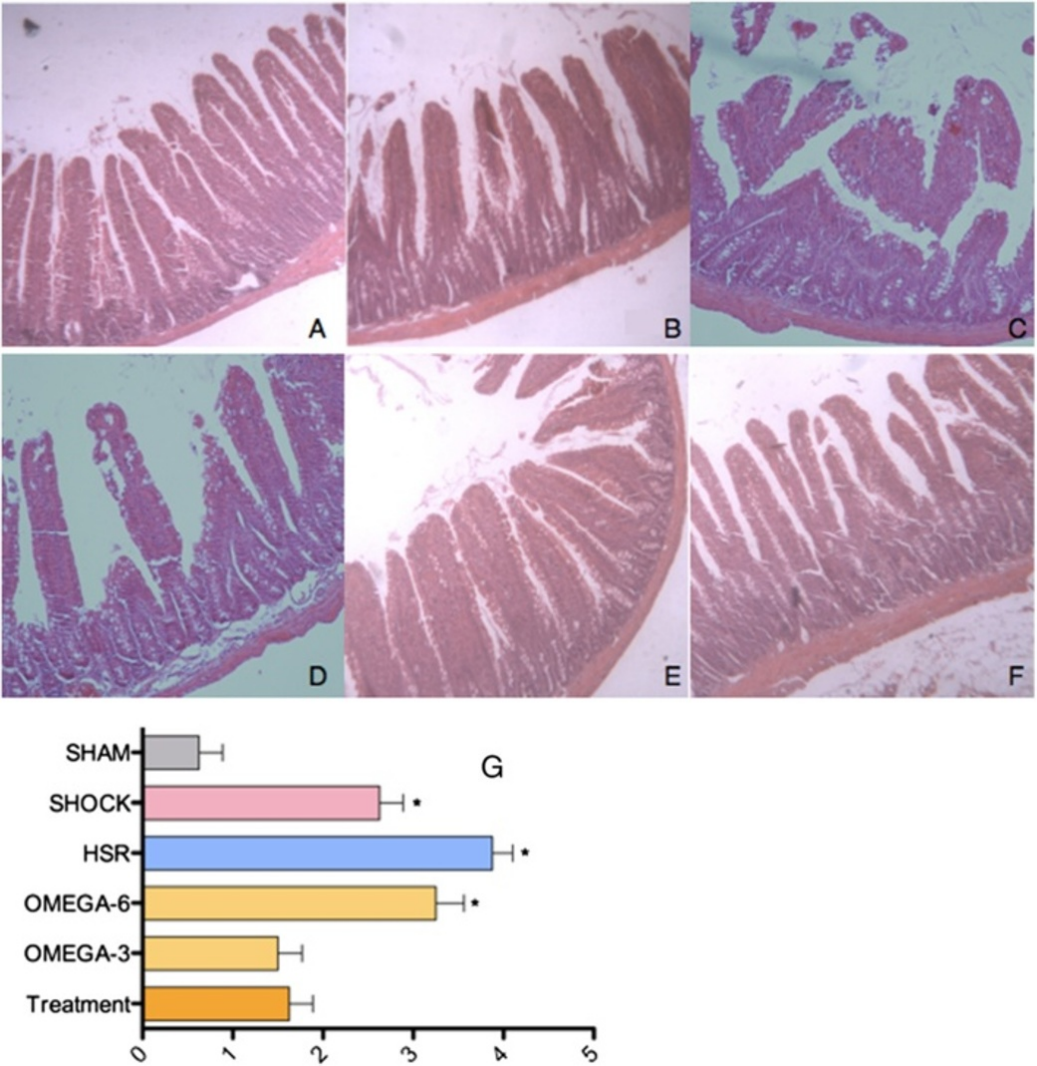
**Histological findings of the jejunum from rats (original magnification, 20 × 20). (A)** Sham animals. **(B)** Hemorrhagic shock. **(C)** Hemorrhagic shock and resuscitation. **(D)** Intralipid infused before resuscitation. **(E)** Fish oil infused before resuscitation. **(F)** Fish oil infused during resuscitation. **(G)** Mucosal damage grading was assessed. The mean histological score of animals in HS group, HSR group and omega-6 group are significantly higher than that of sham mice, and subsequent treatment of ω-3 PUFAs could not attenuate this damage.

Hemorrhagic shock induced the slight intestinal inflammation of the jejunum in the rats (Figure 2B), and the intestine mucosa was destroyed after resuscitation or infused by ω-6 PUFAs (Figure 2C,D). In rats, no matter when injected fish oil, no distinguishable changes of intestinal histopathology were found compared with sham rats (Figure 2E,F). On the basis of a mucosal damage score, infusion of ω-3PUFAs was found to prevent intestinal mucosal damage induced by ischemia/re-perfusion. However, treatment of ω-6PUFAs cannot reverse the effect of ischemia/re-perfusion injury on intestinal mucosa (Figure 2G).

### Tight junctions ultrastructure

**Figure 3:**
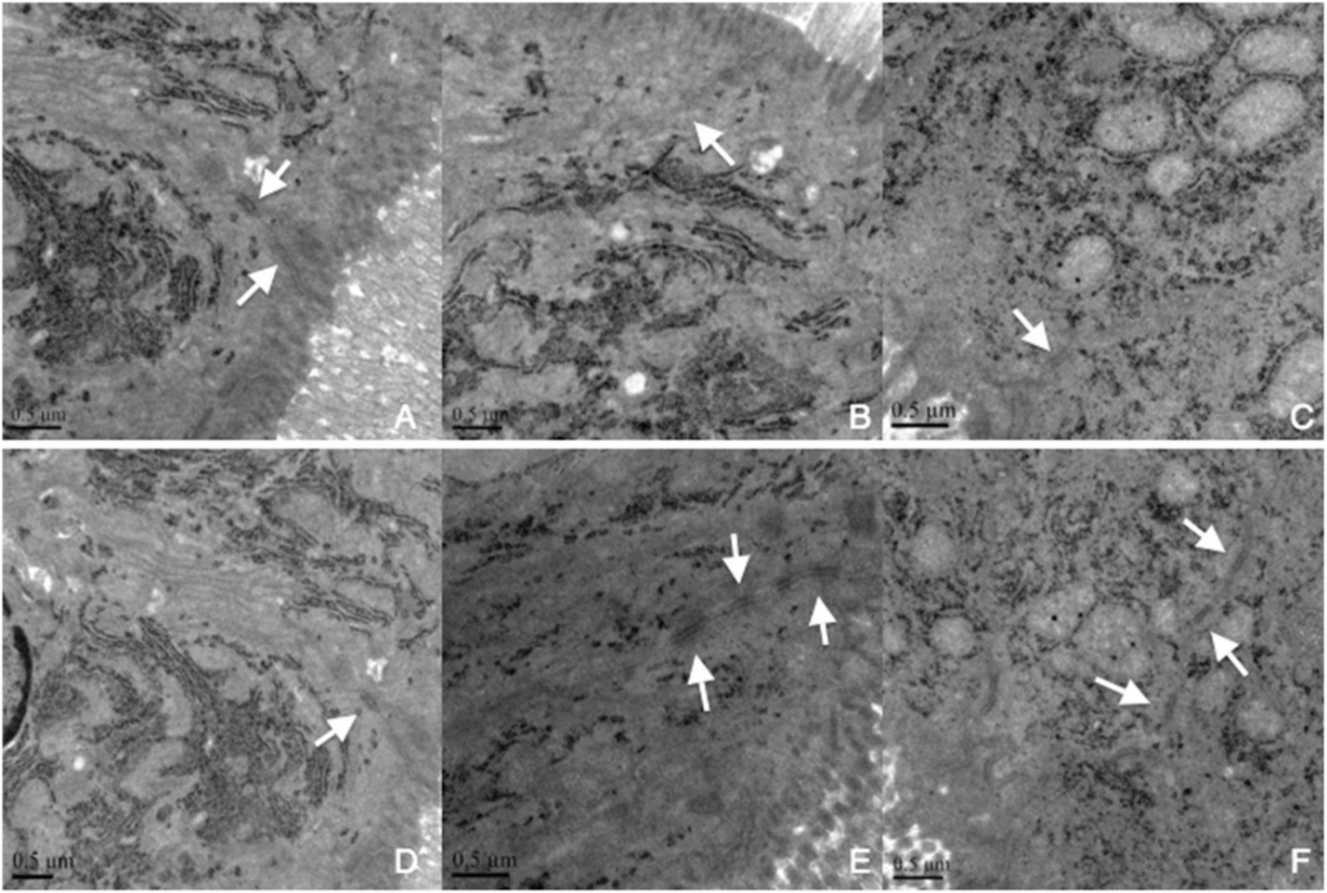
**Tight junction morphology was altered at 4 hours after resuscitation.** The intact TJ structure was observed in sham rats **(A)**. TJ ultrastructure was altered characterized by decreased electron dense materials in the TJ **(B–D)**. TJ ultrastructure was integrity as sham group **(E–F)**. Arrows, tight junctions. Bars = 0.5 μm.

In sham animals, the tight junctions appeared as typical membrane fusions with intact TJs structure (Figure 3A). In contrast, TJs ultrastructure were altered after shock, resuscitation or ω-6PUFAs infusion. Tight junctions were discontinuous with few membrane fusions apparently in colon tissues, which indicated disruption of TJs morphology (Figure 3B-D). There were no morphological differences with the tight junctions in ω-3 group and ω-3 treatment group compared with sham group (Figure 3E-F).

### Redistribution of occludin in epithelium

**Figure 4:**
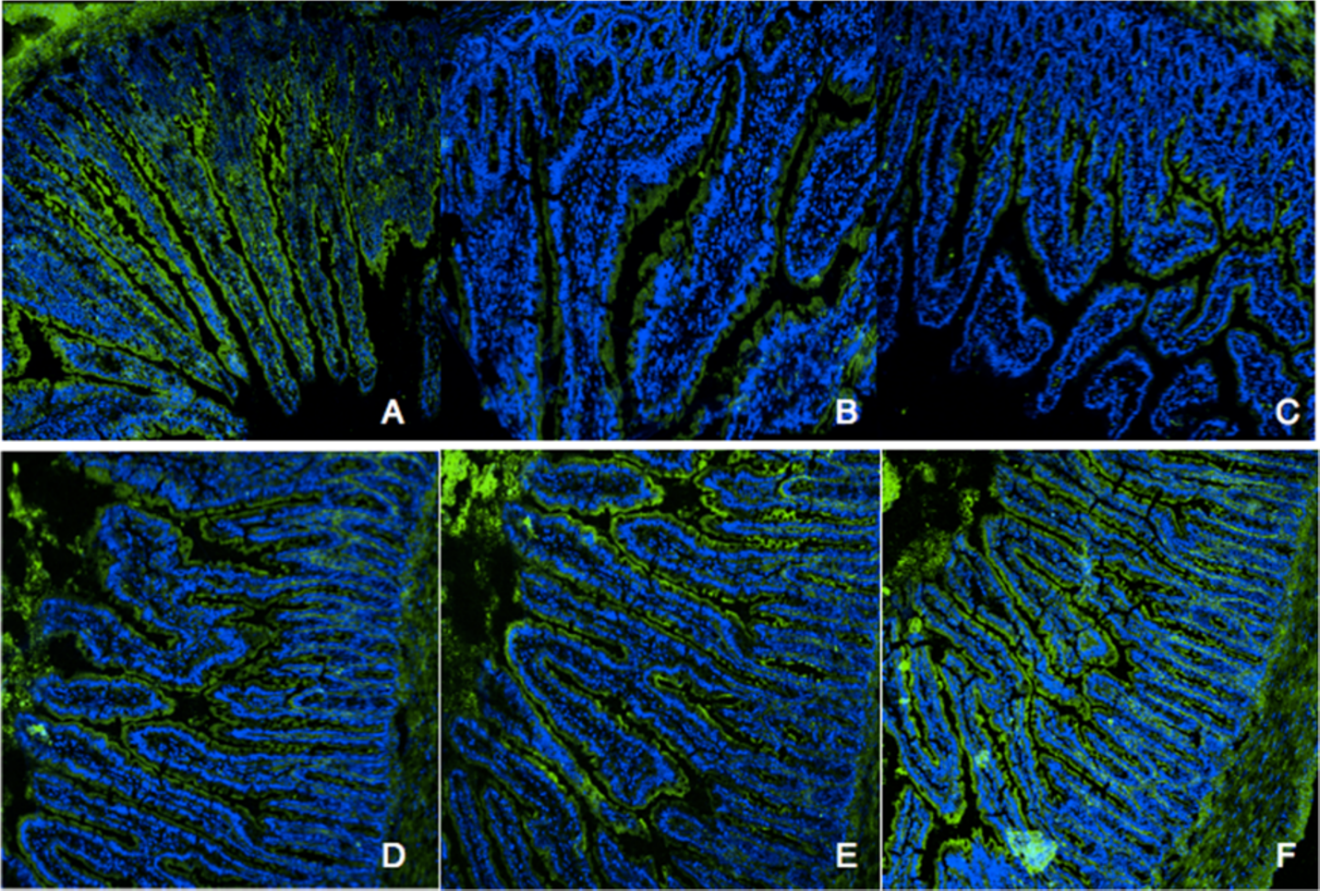
**Redistribution of occludin in hemorrhagic shock rats.** Occludin **(A, E, F)** was precisely localized to the tight junction in the jejunum from sham group, ω-3 group and ω-3 treatment group. In contrast, the jejunum from shock, resuscitation and ω-6 group showed redistribution of occludin **(B, C, D)**. Bars = 25 mm.

Occludin was precisely localized in the villus surface of epithelial cells in jejunum tissues from sham rats (Figure 4A). After shock, resuscitation or intralipid infusion, the distribution of occludin was altered and the epithelium was disrupted (Figure 4B,C,D). Occludin from ω-3 group and ω-3 treatment group had no difference compared with that of sham group.

## Discussion

Hemorrhagic shock followed by resuscitation is known to stimulate a systemic inflammatory response and intestinal barrier dysfunction. Preventing the catastrophic consequences of hemorrhagic shock remains an unsolved problem in clinical treatment of trauma. Daily dietary supplementation with ω-3 PUFAs has been well established as a method to achieve an alleviation of certain acute systemic inflammatory diseases such as asthma, chronic obstructive pulmonary disease, and inflammatory bowel disease [10]. And there have been numerous researches about ω-3 PUFAs in critically ill patients [11, 12]. However, clinical use of ω-3 PUFAs in the systemic inflammatory response syndrome and intestinal barrier function in hemorrhagic shock remains lacking in evidences.

In our study, mucosal Damage Grading Score and H&E-stained sections of jejunum indicated that ω-3 PUFAs alleviates morphological changes induced by ischemia and re-perfusion. The transmission electron microscopy (TME) assay revealed that ω-3 PUFAs extenuated the discontinity and disruption of TJs in the mucosa. By use of immunofluorescence, we demonstrated that ω-3 PUFAs could partially prevent the decreased expression of occludin in jejunum mucosa during hemorrhagic shock followed by resuscitation. These results are similar with other experimental results and the protective effect of fish oil attributed to inflammation reaction alleviating and inflammation factors reducing. Preclinical and clinical research is depicting a therapeutic effect for consumption of the fish-oil derived in the management of inflammatory disease in general [13].

The small intestine is highly sensitive to hypo-perfusion injury because of higher mucosal cell oxygen demand [14, 15]. Thus, subjected to hemorrhagic shock and resuscitation, trauma, and surgery often develop intestinal ischemia and tissue damage, which has been documented by both experimental [16] and clinical studies [17]. In current research, we demonstrated that the administration of ω-3 PUFAs could partly attenuate intestinal epithelial barrier dysfunction in hemorrhagic shock and resuscitation rats. The levels of I-FABP in plasma were significantly decreased after ω-3 PUFAs administration. Our preliminary study showed the intestinal barrier is severely damaged after I/R, which is related to the redistribution of I-FABP and indicated that n-3 PUFAs protect the intestinal barrier by modifying intracellular I-FABP, activating the peroxisome proliferator-activated receptor-γ (PPARγ) pathway, and then upregulating TJs protein expression [18].

In the present study, we found that administration of ω-3 PUFAs regardless of being before or during resuscitation would reduce the systemic inflammation. The levels of IL-6 and TNF-α in intestinal tissue of ω-3 group and ω-3 treatment group were dramatically lower than HS, HSR and ω-6 groups’ (Table 2). This phenomenon may be explained as the effects of ω-3 PUFAs on regulation of host inflammatory responses. And this phenomenon is associated with the change of fatty acids in plasma and epithelial cells (Tables 3 and 4). It is the administration of ω-3 PUFAs that promotes the formation of eicosanoids, which are anti-inflammatory rather than pro-inflammatory mediators. In addition, intravenous infusion of ω-3 PUFAs leads to decreases of prostaglandin E2, thromboxane A2, and leukotriene B4, accompanied with increases of thromboxane A3, prostacyclin PGI3, and leukotriene B5. Generally, the leukotrienes, thromboxanes, and prostaglandins derived from ω-3 PUFAs are less pro-inflammatory than the homologus compounds derived from ω-6 PUFAs. So, the levels of DHA and EPA significantly elevated in plasma in ω-3 group and ω-3 treatment group were the primary cause for less systemic inflammation in the two groups. There are, however, powerful anti-inflammatory metabolites from the ω-6 PUFAs AA, and one cannot say with validity that “ω-6 are pro-inflammatory and ω-3 are not” [19]. So we did not find the inflammatory response becomes worse in the rats of ω-6 group after ω-6 PUFAs infusion.

However, the levels of endotoxin in ω-3 group and ω-3 treatment group were dramatically escalated at 4 hours after resuscitation when compared with them in 30 min. What makes the serum endotoxin levels grow up, furthermore why the elevated endotoxin concentration did not induce more inflammatory response and gut barrier damage? Pluess et al. have domesticated that healthy volunteers received twice 0.5 g/kg fish oil at 48 and 24 h before lipopolysaccharide (LPS) injected would modify the phospholipid composition of platelets and blunted fever and increased the neuroendocrine and the inflammatory responses to LPS [20]. Meanwhile, in their previous study in healthy subjects they showed that prolonged supplementation with oral fish oil blunts the neuroendocrine and inflammatory responses to LPS challenge [21]. Similarly, Pittet et al. have divided male subjects into three groups which received three different fish oil doses before LPS. And the 0.2 g/kg per-fusion immediately (2 hours) before LPS was the most efficient in blunting the responses, suggesting LPS capture in addition to the systemic and membrane effects. These studies may explain the phenomenon found in our experiments, although the concentration of endotoxin magnified, the infusion of fish oil will blunt the inflammatory and neuroendocrine responses to alleviate the damage of gut mucosa.

## Conclusion

In conclusion, our data indicate—to our knowledge—that ω-3 PUFAs can ameliorate intestinal epithelium TJ disruption in a rat model of hemorrhagic shock followed by resuscitation. We speculate that ω-3 PUFAs, individually or in combination with other therapy, may serve as a potential new therapeutic agent that restores the normal intestinal barrier function in hemorrhagic shock.

## Materials and methods

### Animals

Seventy-two male SD rats of a mean (±SD) weight of 269.26 ± 12.68 g were used and the protocol of this study was approved by the institutional animal care committee. Animals, supplied by Medical Experiment Animal Center of Jinling Hospital (Nanjing, China), were kept in a chamber with a light cycle from 9 am to 9 pm and controlled temperature of 25°C–28°C with food and water available ad libitum. Characteristics of the study groups are presented in Table 5.

**Table 5:** **Characteristics of study groups**

SHAM (n = 12)	control (cannulation and Heparinized)
HS (n = 12)	control + shock
HS/R (n = 12)	HS + fluid resuscitation
ω-6 group (n = 12)	HS/R + intralipid (0.2 g/kg) intravenous after shock 30 min
ω-3 group (n = 12)	HS/R + fish oil (0.2 g/kg) intravenous after shock 30 min
ω-3 treatment group (n = 12)	HS/R + fish oil (0.2 g/kg) intravenous during resuscitation

HS, hemorrhagic shock; HS/R, hemorrhagic shock and resuscitation.

### Rat model of hemorrhagic shock and resuscitation

All rats were weighed and anesthetized with 60 mg/kg ketamine intramuscular injection and then placed on a temperature-controlled heating pads, which was heated while shock was induced and temperature was sustained 37°C. Using aseptic techniques, the left femoral artery and vein were isolated and cannulated with a short incision containing 0.1 ml heparinized saline (10 units/ml) [22]. Heparin (200 U/kg) was infused immediately through femoral vein following cannulation. The artery catheter was connected to a multi-channel recorder (Electronics for Medi- cine, TX) for the monitoring of systolic, diastolic, and mean arterial pressures (MAP) per 3 minutes. The target MAP (35 ~ 40 mm Hg) was induced by removing blood slowly through the femoral vein cannula during a total time of 15 minutes and maintained at 35 to 40 mm Hg with further withdrawals of blood as required for 60 minutes. At the point of 75 minutes, resuscitation which would take 30 minutes was implemented by returning half of the shed blood and Ringer’s solution to the animal at the speed of 0.5 mL/min. And Infusion was withheld if MAP exceeded 90% of baseline and re-started if declined. At the 105th minute, resuscitation was finished and no interventions were carried out. The rats in ω-6 group and ω-3 group were injected by intralipid and fish oil (Omegaven, Huarui Pharmaceutical Co., Ltd. 0.2 g/Kg) respectively through the caudal vena after shock 30 min. And the rats in ω-3 treatment group were injected by fish oil (0.2 g/Kg) during resuscitation. At the end of 30 minutes of resuscitation, half of the animals of these three groups were randomly sacrificed to collect blood samples, and the others were killed at the end of 4 hours. The 2–3 ml sample was centrifuged at 4°C. After centrifugation, serum was kept refrigerated at -80°C until assayed.

### Measurement of I-FABP and endotoxin in plasma

The blood sample was centrifuged at 10 000 × g for 15 min at 4°C. The supernatant was assayed for I-FABP and endotoxin levels by enzyme-linked immunosorbent assay kits (R&D Systems, Minneapolis, MN, USA) in accordance with the manufacturer’s instructions. The absorbance at 450 nm was determined using a microplate reader (Tecan’s, Sunrise, Unterberg strasse IA, Austria).

### Measurement of IL-6 and TNF-α in tissue

The jejunum at 2 cm beyond Treitz ligament was weighed and homogenized by adding 9 times gravimetric saline. The 10% homogenate was centrifuged for 10 minutes (2500 r/min) and the supernatant was diluted 10 times with normal saline to 1% concentration. The 1% supernatant was assayed for IL-6 and TNF-a levels by enzyme-linked immunosorbent assay kits (R&D Systems, Minneapolis, MN, USA) in accordance with the manufacturer’s instructions.

### Fatty acid analysised

To verify that the treatments change resulted in the incorporation of significant amounts of DHA and EPA into membrane lipids and plasma, fatty acid analysised was carried out in intestinal epithelial cells and plasma. Small intestine samples were first homogenized and the cell pellet isolated by centrifugation and frozen until analysis. Total small intestine lipids were extracted with methanol and methylene (both containing 50 mg/L BHT) as described [23]. The organic phase was collected and the solvent evaporated under nitrogen in a 45°C water bath. The samples were methylated as described [24]. The samples were analyzed by gas chromatography (GC-14A; Shimadzu, Columbia, MD) using a fused silica capillary column (SP-2560, 100 m; Supelco, Bellefonte, PA). Fatty acid methyl esters are reported as percent of total and were identified by comparison with known standards.

DHA and EPA in plasma were quantified by one-step rapid extractive methylation for gas chromatographic analysis [25], as described previously [26, 27]. First, citrate plasma was spiked with heptadecanoic acid as internal standard. Next, free fatty acids were converted to methyl esters by mixing with ethereal diazomethane. Lastly, the ethereal layer was dried, redissolved in chloroform, and transferred to the gas chromatograph. The fatty acid methyl esters were detected by use of a flame ionization detector, and peak area integration was performed.

### Histopathological evaluation

The proximal jejunum specimens were harvested immediately after the animals were sacrificed, and rinsed in cold saline with subsequent immersion in 10% buffered formalin over- night at room temperature. Sections measuring 5-μm in thickness were cut down, placed on glass slides, and stained with hematoxylin and eosin (H&E). Images were obtained using a Zeiss Image A1 light microscope at × 20 magnification with AxioVision V4.5 software. Three pathologists, blinded to the source of the slides, analyzed and made a report about every slide. The degree of histopathologic changes was graded semiquantitatively by using the histologic injury score described by Chiu et al. [28] (Table 6).

**Table 6:** **Intestinal mucosal damage grading score**

Grade	Histological characteristic(s)
Grade 0	Normal mucosal villi
Grade 1	Subepithelial Gruenhagen’s space (oedema), usually at the apex of the villus
Grade 2	Extension of the subepithelial space with moderate lifting of epithelial layer from the lamina propria
Grade 3	Massive epithelial lifting down the sides of villi; a few tips may be denuded
Grade 4	Denuded villi with lamina propria and dilated capillaries exposed
Grade 5	Digestion and disintegration of lamina propria; haemorrhage and ulceration

### Transmission electron microscopy (TEM)

Two-millimeter sections of colon were fixed for 2 h in 4% buffered glutaraldehyde. The sections were cut into smaller pieces, then fixed by 1% OsO4, sequentially dehydrated through graded alcohols, infiltrated through Epon 812 and then embedded in resin. Thin sections were cut and stained with uranyl acetate and lead citrate, and examined with a Hitachi H-600 (Tokyo, Japan) transmission electron microscope operated at 75 kV.

### Laser scanning confocal microscopy (LSCM)

Immunofluorescence staining of the proximal jejunum tissues was performed according to the method described by Guttman et al. [29]. After being rinsed with ice-cold PBS, tissues were fixed in 3% paraformaldehyde, embedded in an optimal cutting temperature compound (OCT; Sakura Finetech, USA) and 6 mm thick serial sections were cut. Tissue sections were treated with 0.2% Triton X- 100 in phosphate-buffered saline (PBS) for 20 min and then blocked with 5% normal goat serum in PBS containing 0.05% Tween-20 and 0.1% bovine serum albumin. Tissues were incubated with monoclonal antibodies against occludin or ZO-1 (1:200) in PBS with 1% goat serum overnight at 4°C. After washing, sections were incubated with Alexa 488-conjugated secondary antibodies for 60 min. DAPI was used for staining DNA of cells. Images were captured using a Leica TCS SP2 laser confocal scanning microscope (Leica Micro- systems, Heidelberg GmbH, Mannheim, Germany).

### Statistical analysis

Data results are presented as mean ± standard error. Comparisons of various hemorrhagic shock parameters, I-FABP, endotoxin, oxidative and inflammatory molecules, blood, and histological score between the four groups were performed by ANOVA test. A significance level of .05 was used for all analyses.

## Abbreviations

n-3 PUFAs: n-3 polyunsaturated fatty acids
TJ: Tight junction
HS: Hemorrhagic shock
HS/R: Hemorrhagic shock/resuscitation
MAP: Mean arterial pressure
HE: Histologic examination
SEM: Scanning electron microscopy
LSCM: Laser scanning confocal microscope
MODS: Multiple-organ dysfunction syndrome
SIRS: Systemic inflammatory response syndrome
I-FABP: Intestinal fatty acid binding protein
LPS: Lipopolysaccharide.
